# Defining, Locating, and Characterizing Psychiatrists who Primarily Treat Children and Adolescents and their Practices in Ontario: A Cross-Sectional Study: Définir, localiser et caractériser les psychiatres qui traitent principalement les enfants et les adolescents et leurs pratiques en Ontario : étude transversale

**DOI:** 10.1177/07067437251408168

**Published:** 2025-12-29

**Authors:** Madison MacKinnon, Alene Toulany, Claire de Oliveira, Tea Rosic, Paul Kurdyak

**Affiliations:** 1 Institute of Health Policy, Management, and Evaluation, Dalla Lana School of Public Health, University of Toronto, ON, Canada; 2 Institute for Mental Health Policy Research and Campbell Family Mental Health Research Institute, Centre for Addiction and Mental Health, Toronto, ON, Canada; 3 ICES, Toronto, ON, Canada; 4 Department of Paediatrics, Division of Adolescent Medicine, The Hospital for Sick Children, Toronto, ON, Canada; 5 Child Health Evaluative Sciences, SickKids Research Institute, Toronto, ON, Canada; 6 Children's Hospital of Eastern Ontario Research Institute, Ottawa, ON , Canada; 7 Department of Psychiatry, University of Ottawa, Ottawa, ON, Canada

**Keywords:** psychiatrists, children, adolescents, administrative data, Ontario, Psychiatres, enfants, adolescents, données administratives, Ontario

## Abstract

**Objective:**

The current supply and distribution of child psychiatrists in Ontario is not well understood, making it difficult to effectively plan mental healthcare services for children and adolescents. Therefore, we developed a data-driven definition of psychiatrists who focus on treating child and adolescents, and described their demographic characteristics, geographic distribution, and practice patterns across Ontario in 2023.

**Method:**

A cross-sectional study was employed using administrative data from ICES. All practicing Ontario-based psychiatrists, defined as those submitting at least one billing claim to the Ontario Health Insurance Plan were included. Psychiatrists from the years 2013–2023 were included to create the definition of child-focused psychiatrists. Child-focused psychiatrists were defined as those with ≥50% or more of their patients ≤18 years of age. Then, this definition was applied to psychiatrists in 2023 to compare and descriptively summarize data (e.g., age, sex, rurality of practice location, and practice patterns) between child- and adult-focused psychiatrists.

**Results:**

In 2023, there was a total of 259 child-focused psychiatrists and 2,099 adult-focused psychiatrists in Ontario. Child-focused psychiatrists were younger (mean age ± SD: 55.8 ± 9.3 vs. 60.1 ± 11.5, p < 0.001), more likely to be female (59.1% vs. 46.2%, p < 0.001), and less likely to work in rural regions than adult-focused psychiatrists. Both, on average, saw a similar number of patients overall (276.7 ± 265.9 vs. 329.3 ± 403.1, p = 0.115), but child-focused psychiatrists saw patients less frequently than adult-focused psychiatrists (3.0 ± 1.8 vs 6.5 ± 9.1, p<0.001). Child-focused psychiatrists were less likely to have small patient panels as well (p < 0.001).

**Conclusions:**

Child-focused psychiatrists represent a small proportion of the psychiatric workforce in Ontario, with particularly limited availability in rural regions. Compared to adult-focused psychiatrists, they are less likely to maintain smaller practices and they see their patients less frequently.

## Résumé

**Objectif:**

L’offre et la répartition actuelles des pédopsychiatres en Ontario ne sont pas bien comprises, ce qui rend difficile la planification efficace des services de santé mentale pour les enfants et les adolescents. Par conséquent, nous avons élaboré une définition fondée sur les données des psychiatres qui se concentrent sur le traitement des enfants et des adolescents et nous avons décrit leurs caractéristiques démographiques, leur répartition géographique et leurs habitudes de pratique à l’échelle de l’Ontario en 2023.

**Méthode:**

Une étude transversale a été réalisée à l’aide des données administratives de l’ICES. Tous les psychiatres actifs en Ontario, c’est-à-dire ceux qui ont présenté au moins une demande de facturation à l’Assurance-santé de l’Ontario, ont été pris en compte. Les années 2013 à 2023 ont été utilisées pour créer la définition des psychiatres qui se concentrent sur les enfants. Ces derniers ont été définis comme des psychiatres dont au moins 50 % des patients avaient ≤ 18 ans. Cette définition a ensuite été appliquée aux psychiatres en 2023 pour comparer et résumer les données de façon descriptive (p. ex., âge, sexe, ruralité du lieu de pratique et tendances de pratique) entre les psychiatres qui se concentrent sur les enfants et les adultes.

**Résultats:**

En 2023, l’Ontario comptait un total de 259 psychiatres qui se concentrent sur les enfants et de 2 099 psychiatres qui se concentrent sur les adultes. Les psychiatres qui se concentrent sur les enfants étaient plus jeunes (âge moyen ± É.-T. : 55,8 ± 9,34 par rapport à 60,1 ± 11,5; p < 0,001), avaient plus tendance à être des femmes (59,1 % par rapport à 46,2 %; p < 0,001) et avaient moins tendance à travailler dans les régions rurales que les psychiatres qui se concentrent sur les adultes. Dans les deux cas, en moyenne, le nombre de patients était semblable dans l’ensemble (276,7 ± 265,9 c. 329,3 ± 403,1; p = 0,115), mais les psychiatres qui se concentrent sur les enfants voyaient les patients moins souvent que les psychiatres qui se concentrent sur les adultes (3,02 ± 1,79 c. 6,51 ± 9,07). Les psychiatres qui se concentrent sur les enfants avaient également moins tendance à avoir de petits groupes de patients (p < 0,001).

**Conclusions:**

Les psychiatres qui se concentrent sur les enfants représentent une faible proportion de l’effectif psychiatrique en Ontario, et leur disponibilité est particulièrement limitée dans les régions rurales. Comparativement aux psychiatres qui se concentrent sur les adultes, ils ont moins tendance à maintenir des pratiques plus modestes et à voir leurs patients moins souvent.

## Introduction

Adolescents are an at-risk population for the development of mental illness.^1–3^ Despite this, fewer than 20% of children and adolescents with mental illness receive appropriate treatment.^4^ Psychiatrists play an important role in delivering mental healthcare to children and adolescents, particularly in managing complex and/or severe cases. The Canadian Psychiatric Association recommends a supply of 15 psychiatrists per 100,000 population,^5^ and the Canadian Academy of Child and Adolescent Psychiatry recommends one child psychiatrist for every 4,000 youth aged 19 and younger.^6,7^ Child psychiatrists often lead interdisciplinary mental healthcare teams that may include psychiatric nurses, social workers, psychologists, therapists, and other mental health professionals.^8,9^

Despite child psychiatry being an accredited subspecialty, there are limited data on the practice patterns and characteristics of child psychiatrists in Canada. Most Canadian studies examining psychiatric care have focused on the barriers to access and the overall low supply of psychiatrists across the country.^6,10–14^ Only one study by Steele and Wolfe^15^ investigated the demographics and practice patterns of child psychiatrists in Ontario. They found that only 38.9% of child psychiatrists devoted the majority of their clinical time (75–100%) to children and adolescents, while 20.3% spent 25% or less of their time with this population.^15^ However, these findings are based on data from 1997 and are unlikely to reflect the current practice patterns or workforce distribution. Two more recent Canadian studies^5,16^ have used administrative data to assess psychiatrist's characteristics, practice patterns, and geographic distribution in Ontario, but they did not examine child psychiatrists specifically.

The limited data on child psychiatrists may, in part, reflect the absence of a clear operational definition for this subspecialty. A child psychiatrist may not care exclusively for patients under the age of 18. Although the Royal College of Physicians and Surgeons of Canada has established formal training pathways and competencies for child psychiatry,^17^ some psychiatrists provide care to children and adolescents without formal training, often due to the shortage of child psychiatrists.^15^ As such, a data-driven definition based on the proportion of paediatric patients seen may offer a more accurate and practical approach to identifying those psychiatrists who primarily treat children and adolescents, recognizing that formal training alone may not imply that a psychiatrist will only treat these populations.

A comprehensive summary of the characteristics and location of practice of psychiatrists who primarily treat children and adolescents (hereafter referred to as “child-focused psychiatrists”), using a data-driven definition of child-focused psychiatrists is needed to understand the true supply and distribution of psychiatric care for children and adolescents in Ontario. Our aim was to identify the current supply of child-focused psychiatrists, their practice patterns, and regional distributions. Specifically, we aimed to first develop a data-driven definition of child-focused psychiatrists and then geographically locate and characterize child-focused psychiatrists and their practices in Ontario in 2023 compared to adult-focused psychiatrists.

## Materials & Methods

### Study Design & Setting

A cross-sectional descriptive study in Ontario, Canada was employed, using administrative data obtained from ICES. ICES is an independant, non-profit research institute whose legal status under Ontario's health information privacy law allows it to collect and analyze health care and demographic data, without consent, for health system evaluation and improvement. The use of these data is authorized under section 45 of Ontario's Personal Health Information Protection Act (PHIPA) and does not require review by a Research Ethics Board. Data from 2013–2023 were used to create the definition of child-focused psychiatrists. Data from 2023 were collected on psychiatrist characteristics, practice patterns, and patient demographics.

### Data Sources

Various ICES databases were accessed to undertake this study. The Ontario Health Insurance Plan (OHIP) database was accessed to identify our study population in the years 2013–2023. Psychiatrist characteristics were collected from the ICES Physician Database (IPDB) and the Corporate Provider Database (CPDB), and practice characteristics were collected from the OHIP database, which captures the location of physician care (inpatient, office, or over the phone). Patient characteristics were collected from the Registered Persons Database (RPDB), the Ontario Mental Health Reporting System (OHMRS) for inpatient psychiatric admissions, the Discharge Abstract Database (DAD) for child and youth psychiatric and non-psychiatric hospital admissions, the National Ambulatory Care Reporting System (NACRS) for emergency department (ED) care, and OHIP claims for diagnosis. These datasets were linked using unique encoded identifiers and analysed at ICES. Population data identifying the number of youth in Ontario for per capita calculations were obtained from Statistics Canada.^18^

### Population

This study included all psychiatrists practising in Ontario, identified by those who submitted at least one billing claim to OHIP during the study period. Providers who do not submit billings through OHIP were excluded. Practicing psychiatrists from 2013 to 2023 were included to create the definition of child-focused psychiatrists. This definition was then applied to practicing psychiatrists in 2023 to identify child-focused and adult-focused psychiatrists in this year and analyse their characteristics and practice patterns.

### Child-Focused Psychiatrist Definition

To define a child-focused psychiatrist, we first examined the distribution of the proportion of paediatric patients seen by psychiatrists and then identified an appropriate cut-off based on this proportion. Initially, we considered paediatric patients as those <18 years of age in keeping with the legal age of adulthood in Ontario^19^ and the provincially mandated age of transfer from child to adult health services at 18 years.^20^ However, based on clinical experience, the transfer process to adult care often extends into the 18^th^ year,^20^ with many adolescents continuing to receive care from paediatric providers after their 18^th^ birthday. Therefore, we defined paediatric patients as those who were ≤18 years of age. After reviewing the proportion of individuals aged ≤18 years seen in both outpatient and inpatient settings, psychiatrists were classified as child-focused psychiatrists if ≥50% of their patient encounters were with individuals ≤18 years of age.

### Psychiatrist & Patient Characteristics

Characteristics of psychiatrists examined included age, sex, practice location, and years since medical school graduation. Practice location was defined by two measures, the former Local Health Integration Network (LHIN) which was made up of 14 local health care systems across Ontario and rurality, identified by Rurality Index for Ontario (RIO) score.^21,22^ In 2021, LHINs were replaced by Ontario Health (OH) Regions to improve integrated care across the province. We used LHINs as they provide a more detailed comparison across the province than OH Regions. We subcategorized LHINs into high-supply (Toronto Central, Champlain, and South East), medium-supply (South West, North West, and Hamilton Niagara Haldimand Brant), and low-supply LHINs (Central, Central East, Central West, Eerie St. Clair, Mississauga Halton, North East, North Simcoe Muskoka, Waterloo Wellington) following an approach used in other similar work.^23^ LHINs were categorized based on whether their average supply per capita of psychiatrists was much higher (high-supply), at or near (medium-supply), or much lower (low-supply) than the provincial average.^23^ The RIO score, developed by the Government of Ontario, is a measure of rurality that is derived from a community's population and travel time to a basic or advanced referral centre.^21^ Higher scores indicate more rural communities; scores of 0–39 were considered urban and scores of ≥40 were considered rural, as in previous studies.^24,25^

Psychiatrist practice characteristics examined included the frequency of patient diagnoses (anxiety disorder, behavioural disorder, mood disorder, personality disorder, psychotic disorder, substance use disorder, other based on OHIP claims), number of patients seen per year (overall, outpatients, inpatients and new inpatients and outpatients, defined as patients seen in 2023 who were not seen in the previous year by that psychiatrist) on average and categorized, number of outpatient visits per patient per year, on average and categorized as <4 visits, 4–12 visits, > 12 visits. Patient characteristics examined included age, sex, location (using the RIO score), neighbourhood-level income expressed as quintiles (used a proxy for individual or household income), mental illness diagnosis (psychotic disorder, non-psychotic disorder, substance use, and other based on OHIP claims), outpatient visit frequency, and health service use such as hospitalizations (both mental health-related and total) and ED visits (both mental health-related and total).

### Data Analysis

Child-focused psychiatrist characteristics, practice patterns, and respective patient characteristics were summarized descriptively and compared to adult-focused psychiatrists, using means and standard deviations for continuous measures and frequency and proportions for categorical measures. The per capita supply of child-focused psychiatrists among children in Ontario and number of children and adolescents per child-focused psychiatrist were calculated. Characteristics were assessed for normality where applicable and t-tests, Wilcoxon rank sum test, Kruskal–Walis test, ANOVA and chi-square tests were calculated to compare characteristics between the two groups and between LHIN supply categories.

Various sensitivity analyses were conducted on our definition of a child-focused psychiatrist. The age range of youth includes those through the age of 24; therefore, we conducted a sensitivity analysis considering “paediatric patients” as those <24 years of age. We also conducted a sensitivity analysis defining child-focused psychiatrists as those with ≥30% and ≥70% of their cases ≤18 years of age. All analyses were conducted on R Studio^26^ and were conducted at ICES. A p-value of 0.05 was the cut-off for statistical significance for all tests.

## Results

### Child-Focused Psychiatrist Supply

Based on our definition, there was a total of 259 child-focused psychiatrists and 2,099 adult-focused psychiatrists in Ontario in 2023. The supply of child-focused psychiatrists in the province amounts to 8.0 child-focused psychiatrists per 100,000 children and adolescents or 1 child-focused psychiatrist for every 12,442 children and adolescents (ages 0–19).

### Child-Focused Psychiatrist Characteristics in 2023

Child-focused psychiatrists were primarily female (59.1% vs 46.2%, p < 0.001), younger (55.8 ± 9.3 years vs 60.1 ± 11.5 years (mean ± SD), p < 0.001), and were less likely to practice in rural areas (1.8 ± 4.2 RIO score vs 3.1 ± 9.0 RIO score, p = 0.022) compared to adult-focused psychiatrists (Table 1).

**Table 1.** Child- and Adult-Focused Psychiatrist Demographics in 2023.

	Child-focused psychiatrists	Adult-focused psychiatrists	p-value
n	259	2099	
**Age (mean (SD))**	55.8 (9.3)	60.1 (11.5)	<0.001
**Sex (n(%))**			<0.001
*Female*	153 (59.1)	970 (46.2)	
*Male*	106 (40.9)	1129 (53.8)	
**Canadian medical graduate - Yes (n(%))**	99 (65.6)	850 (63.0)	0.597
**Years since medical school graduation (mean (SD)**	24.7 (11.4)	28.5 (14.3)	<0.001
**Years since medical school graduation (n(%))**			<0.001
*0–15 years*	85 (32.8)	596 (28.4)	
*15–30 years*	110 (42.5)	692 (33.0)	
*30+ years*	64 (24.7)	811 (38.6)	
**Practice location - LHIN (n(%))^a^**			0.217
*Central*	17 (6.6)	157 (7.5)	
*Central East*	15 (5.8)	97 (4.6)	
*Central West*	8 (3.1)	36 (1.7)	
*Champlain*	28 (10.8)	238 (11.3)	
*Erie St. Clair*	8 (3.1)	44 (2.1)	
*Hamilton Niagara Haldimand*	22 (8.5)	160 (7.6)	
*Mississauga Halton*	16 (6.2)	105 (5.0)	
*North East*	7 (2.7)	44 (2.1)	
*North Simcoe Muskoka*^b^	<6	36 (1.7)	
*North West*^b^	<6	9 (1.4)	
*South East*^b^	<6	89 (4.2)	
*South West*	19 (7.3)	125 (6.0)	
*Toronto Central*	62 (23.9)	666 (31.7)	
*Waterloo Wellington*	12 (4.6)	79 (3.8)	
**Practice location - RIO score (mean (SD))**	1.8 (4.2)	3.1 (9.0)	0.022
**Practice location - RIO score categories (n(%))**			0.189
*0 (Urban)*	166 (73.1)	1418 (74.2)	
*1–39 (Urban)*	61 (26.9)	458 (24.0)	
*40–80 (Rural)*	0 (0.0)	34 (1.8)	

^a^ Missing data for n = 33 child and n = 194 adult-focused psychiatrists.

^b^ Cells collapsed due to small cell counts.

LHIN = Local Health Integration Network; RIO = Rurality Index of Ontario; SD = standard deviation.

While, on average, both groups saw a similar number of patients overall, adult-focused psychiatrists saw, on average, significantly more inpatients and new inpatients compared to child-focused psychiatrists (p = 0.001) (Table 2). Although the subgroups did not significantly differ on the average number of outpatients seen, child-focused psychiatrists saw significantly more new outpatients (p = 0.02). Child-focused psychiatrists were also less likely to have small outpatient panels (p < 0.001). Adult-focused psychiatrists saw their outpatients more often; they averaged approximately seven visits per outpatient per year compared to approximately three visits for child-focused psychiatrists (p < 0.001).

**Table 2.** Child- and Adult-Focused Psychiatrist Practice Characteristics in 2023.

	Child-focused psychiatrists	Adult-focused psychiatrist	p-value
n	259	2099	
**Patients seen overall**			
*Mean (SD)*	276.7 (265.9)	329.3 (403.1)	0.115
*Median (range)*	195 (1–1789)	209 (1–7403)	0.71
**No. of inpatients seen**			
*Mean (SD)*	53.4 (75.8)	81.5 (136.8)	0.001
*0 patients (%)*	57 (22.0)	831 (39.6)	<0.001
*1–50 patients (%)*	116 (44.7)	494 (23.4)	
*51–100 patients (%)*	39 (15.1)	207 (9.9)	
*>100 patients (%)*	47 (18.1)	567 (27.0)	
**No. of new inpatients seen**			
*Mean (SD)*	48.8 (67.5)	73.9 (122.2)	0.001
*0 patients (%)*	57 (22.0)	838 (39.9)	<0.001
*1–50 patients (%)*	118 (45.6)	511 (24.3)	
*51–100 patients (%)*	42 (16.2)	207 (9.9)	
*>100 patients (%)*	42 (16.2)	543 (25.9)	
**No. of outpatients seen**			
*Mean (SD)*	213.8 (232.4)	222.95 (282.3)	0.616
*0 patients (%)*^a^	<6	24 (1.1)	<0.001
*1–50 patients (%)*^a^	29–39	494 (23.5)	
*51–100 patients (%)*	64 (24.7)	354 (16.9)	
*>100 patients (%)*	162 (62.5)	1227 (58.5)	
**No. of new outpatients seen**			
*Mean (SD)*	140.18 (155.3)	128.98 (191.8)	0.02
*0 patients (%)*^a^	<6	91 (4.3)	<0.001
*1–50 patients (%)*^a^	59–69	821 (39.1)	
*51–100 patients (%)*	81 (31.3)	384 (18.3)	
*>100 patients (%)*	116 (44.8)	803 (38.3)	
**Average no. of visits per outpatient (mean, SD)**	3.0 (1.8)	6.5 (9.1)	<0.001
**Average no. of visits per outpatient, categorized (n(%))**			<0.001
*<4 visits*	204 (79.1)	1179 (56.8)	
*4–12 visits* ^a^	49–59	624 (30.1)	
*>12 visits* ^a^	<6	272 (13.1)	
**Total visits by diagnosis (n(%))**			<0.001
*Anxiety disorder*	94,551 (37.5)	961,422 (35.4)	
*Behavioural disorder*	19,054 (7.6)	13,962 (0.5)	
*Mood disorder*	32,617 (12.9)	360,249 (13.3)	
*Other*	63,247 (25.1)	334,135 (12.3)	
*Personality disorder*	7,835 (3.1)	91,918 (3.4)	
*Psychotic disorder*	32,822 (13.0)	798,288 (29.4)	
*Substance use disorder*	1,753 (0.7)	152,932 (5.6)	

^a^ Cells collapsed due to small cell counts.

### Regional Comparison

In 2023, child-focused psychiatrists in LHINs that had a per capita supply of psychiatrists well under the provincial average per capita supply (low supply) saw, on average, more patients overall (p < 0.001) and more outpatients (p < 0.001) compared to LHINs that had a per capita supply close to (medium supply) or higher than (high supply) the provincial average per capita supply of psychiatrists, respectively. Child-focused psychiatrists in low-supply LHINs were the most likely to see >100 outpatients (83%) compared to medium-supply (69%) and high-supply LHINs (47%) (p < 0.001) and averaged fewer visits per outpatient in the year than high-supply LHINs (2.9 ± 1.8 vs. 2.8 ± 1.6 vs. 3.5 ± 1.9, p < 0.001). These patterns were consistent for adult-focused psychiatrists as well (Table 3).

**Table 3.** Psychiatrist Demographics and Practice Characteristics by LHIN Supply Area Type in 2023.

	Child-focused psychiatrist				Adult-focused psychiatrist			
	Low-supply areas	Medium-supply areas	High-supply areas	p-value	Low-supply areas	Medium-supply areas	High-supply areas	p-value
**n** ^a^	**86**	**45**	**95**		**598**	**314**	**993**	
**Sex (n(%))**				0.07				0.01
*Female*	44 (51.2)	25 (55.6)	66 (69.5)		242 (40.5)	150 (47.8)	485 (48.8)	
*Male*	42 (48.8)	20 (44.4)	29 (30.5)		356 (59.5)	164 (52.2)	508 (51.2)	
**Age (mean (SD))**	55.8 (9.9)	54.5 (8.3)	56.4 (9.4)	0.61	60.2 (11.7)	59.3 (10.9)	60.2 (11.5)	0.74
**Years since graduation (mean (SD))**	24.5 (11.2)	23.4 (10.7)	25.7 (11.8)	0.37	28.8 (14.4)	27.3 (13.7)	28.7 (14.4)	0.46
**Years since graduation (n(%))**				<0.001				<0.001
*0–15*	19 (22.1)	11 (24.4)	22 (23.2)		125 (20.9)	72 (22.9)	210 (21.1)	
*15–30*	45 (52.3)	25 (55.6)	40 (42.1)		218 (36.5)	120 (38.2)	352 (35.4)	
*>30*	22 (25.6)	9 (20.0)	33 (34.7)		255 (42.6)	122 (38.9)	431 (43.4)	
**No. patients overall**								
*Mean (SD)*	404.6 (326.0)	296.7 (254.0)	187.0 (185.8)	<0.001	464.1 (533.3)	360.5 (394.8)	254.5 (302.1)	<0.001
*Median (range)*	313 (20–1798)	234 (7–1298)	130 (1–1165)		332 (1–7403)	244 (1–2800)	175 (1–3942)	<0.001
**No. of outpatients**								
*Mean (SD)*	324.3 (289.4)	242.7 (258.0)	131.0 (123.8)	<0.001	306.9 (320.3)	247.5 (309.0)	178.3 (245.6)	<0.001
*0 (%)*^b^	<6	<6	<6	<0.001	<6	<6	12 (1.2)	<0.001
*1–50 (%)*^b^	<6	<6	9–19		89–99	69–79	262 (26.4)	
*51–100(%)*	10 (11.6)	9 (20.0)	35 (36.8)		80 (13.4)	42 (13.4)	195 (19.6)	
*>100(%)*	71 (82.6)	31 (68.9)	45 (47.4)		423 (70.7)	196 (62.4)	524 (52.8)	
**Average no. of visits per outpatient (mean (SD))**	2.9 (1.8)	2.8 (1.6)	3.5 (1.9)	<0.001	5.0 (5.5)	4.9(5.3)	8.6 (11.7)	<0.001
**Average no. of visits per outpatient**				0.01				<0.001
*<4*	72 (83.7)	38 (84.4)	66 (70.2)		367 (61.8)	190 (61.3)	464 (47.3)	
*4–12* ^b^	9–19	7 (15.5)	28 (30.0)		187 (31.5)	90 (29.0)	323 (32.9)	
*>12* ^b^	<6	0	0		40 (6.7)	30 (9.7)	194 (19.8)	
**No. of new outpatients**								
*Mean (SD)*	194.9 (164.9)	169.4 (215.3)	86.8 (100.4)	<0.001	158.1 (201.7)	132.1 (173.4)	108.9 (187.0)	<0.001
*0 (%)*^b^	0 (0.0)	0 (0.0)	<6	<0.001	18 (3.0)	20 (6.4)	49 (4.9)	<0.001
*1–50 (%)*^b^	10 (11.6)	9 (20.0)	29–39		175 (29.3)	110 (35.0)	461 (46.4)	
*51–100 (%)*	18 (20.9)	13 (28.9)	39 (41.1)		118 (19.7)	53 (16.9)	174 (17.5)	
*>100 (%)*	58 (67.4)	23 (51.1)	21 (22.1)		287 (48.0)	131 (41.7)	309 (31.1)	
**No. of inpatients**								
*Mean (SD)*	79.7 (108.7)	55.8 (64.2)	32.8 (34.6)	<0.001	109.8 (160.5)	97.0 (183.9)	60.8 (99.8)	<0.001
*0 (%)*	24 (27.9)	10 (22.2)	19 (20.0)	<0.001	229 (38.3)	130 (41.4)	424 (42.7)	<0.001
*1–50 (%)*	27 (31.4)	17 (37.8)	51 (53.7)		110 (18.4)	80 (25.5)	239 (24.1)	
*51–100 (%)*^b^	10 (11.6)	7 (15.6)	19–29		48 (8.0)	21 (6.7)	105 (10.6)	
*>100 (%)*^b^	25 (29.1)	11 (24.4)	<6		211 (35.3)	83 (26.4)	225 (22.7)	
**No. of new inpatients**								
*Mean (SD)*	71.4 (96.1)	50.9 (57.4)	30.5 (31.7)	0.001	98.5 (142.5)	85.0 (157.3)	56.0 (93.2)	<0.001
*0 (%)*	24 (27.9)	10 (22.2)	19 (20.0)	<0.001	232 (38.8)	131 (41.7)	427 (43.0)	
*1–50 (%)*	27 (31.4)	17 (37.8)	53 (55.8)		111 (18.6)	79 (25.2)	254 (25.6)	
*51–100 (%)*^b^	11 (12.8)	10 (22.2)	19–29		47 (7.9)	26 (8.3)	103 (10.4)	
*>100 (%)*^b^	24 (27.9)	8 (17.8)	<6		208 (34.8)	78 (24.8)	209 (21.0)	

^a^ Total number of psychiatrists do not add up to 259 and 2099 for child- and adult-focused respectively due to missing LHIN information. LHIN = Local Health Integration Network.

^b^ Cells collapsed due to small cell counts.

Thirty-three child and 194 adult-focused psychiatrists were excluded from the regional analysis due to missing data identifying the LHIN where they practiced. Compared to included psychiatrists, excluded psychiatrists were significantly more likely to be within 15 years from medical school graduation (p < 0.001) and saw, on average, significantly less patients overall and less outpatients for both child- and adult-focused psychiatrists (Supplement).

### Patient Characteristics

Patients who saw child-focused psychiatrists were younger (15.8 ± 9.6 vs 43.6 ± 18.5, p < 0.001) and more likely to be female (59.0% vs. 53.9%, p < 0.001). Patients who saw a child-focused psychiatrist were more likely to see their psychiatrists for fewer visits per year compared to patients who saw adult-focused psychiatrists (4.3 ± 5.6 vs. 6.3 ± 9.7, p < 0.001) (Table 4).

**Table 4.** Characteristics of Patients Who Saw Child and Adult-Focused Psychiatrists in 2023.

	Child-focused psychiatrist	Adult-focused psychiatrist	p-value
**n**	64,532	608,560	
**Sex (n(%))**			<0.001
*Male*	26,488 (41.0)	280,707 (46.1)	
*Female*	38,044 (59.0)	327,853 (53.9)	
**Age (mean(SD))**	15.8 (9.6)	43.6 (18.5)	<0.001
**Age (n(%))**			<0.001
* 0–9*	8,580 (13.3)	1,782 (0.3)	
* 10–19*	48,866 (75.7)	39,503 (6.5)	
* 20–29*	3,387 (5.2)	124,334 (20.4)	
* 30–39*	1,314 (2.0)	124,012 (20.4)	
* 40–49*	830 (1.3)	96,088 (15.8)	
* 50–59*	657 (1.0)	89,959 (14.8)	
* 60–69*	520 (0.8)	70,102 (11.5)	
*>* *70*	378 (0.6)	62,780 (10.3)	
**Income Quintile (n(%))** ^a^			<0.001
*1 (lowest)*	12,884 (20.0)	166,307 (27.3)	
* 2*	12,255 (19.0)	125,904 (20.7)	
*3*	12,210 (18.9)	108,238 (17.8)	
*4*	12,562 (19.5)	99,426 (16.3)	
*5 (highest)*	13,924 (21.6)	101,796 (16.7)	
**RIO score (mean(SD))**	8.29 (13.7)	7.51 (14.2)	<0.001
**RIO category (n(%))**			<0.001
*0 (Urban)*	26,047 (41.1)	313,838 (52.5)	
*1–39 (Urban)*	34,327 (54.1)	253,568 (42.4)	
*40–79 (Rural)*	2,899 (4.6)	28,605 (4.8)	
*Over 80 (Rural)*	149 (0.2)	2,264 (0.4)	
**Number of psychiatric outpatient visits (mean (SD))** ^b^	4.3 (5.6)	6.3 (9.7)	<0.001
**No. outpatient psychiatric visits (n(%))**			<0.001
*0 visits*	2,103 (3.3)	49,167 (8.1)	
*1 visit*	20,561 (31.9)	152,650 (25.1)	
*2–3 visits*	17,857 (27.7)	141,225 (23.2)	
*4–12 visits*	20,411 (31.6)	199,319 (32.8)	
*>12 visits*	3,600 (5.6)	66,199 (10.9)	
**Psychiatric visits by disorder (mean(SD))** ^c^			
*Psychotic disorder*	1.94 (9.9)	6.54 (19.7)	<0.001
*Non-psychotic disorder*	4.70 (11.5)	5.99 (13.6)	<0.001
*Substance abuse*	0.13 (1.5)	0.88 (4.8)	<0.001
*Other*	1.44 (6.2)	0.47 (3.2)	<0.001
**Number of hospitalizations (all) (mean(SD))** ^d^	1.85 (1.8)	1.99 (2.0)	<0.001
**Number of MHA hospitalizations (mean(SD)** ^)d^	1.54 (1.7)	1.41 (1.8)	<0.001
**Number of ED Visits (all) (mean(SD))** ^e^	3.21 (5.2)	4.36 (8.8)	<0.001
**Number of MHA ED visits (mean(SD))** ^e^	1.29 (2.7)	1.74 (4.3)	<0.001
**Hospitalizations (all)** **=** **Yes (%)**	19,890 (30.8)	228,616 (37.6)	<0.001
**MHA hospitalizations = Yes (%)**	17,374 (26.9)	172,509 (28.3)	<0.001
**ED visits (all)** **=** **Yes (%)**	35,821 (55.5)	355,531 (58.4)	<0.001
**MHA ED visits = Yes (%)**	20,360 (31.6)	196,166 (32.2)	<0.001

^a^ Missing data for n = 697 and n = 6889 patients of child and adult-focused psychiatrists, respectively.

^b^ Only those individuals who used had at least one outpatient visit in 2023.

^c^ Calculations include inpatient and outpatient visits.

^d^ Only those individuals who used had at least one hospitalization in 2023.

^e^ Only those individuals who used had at least one ED visit in 2023.

ED = Emergency department; LHIN = Local Health Integration Network; MHA = Mental Health and Addiction; RIO = Rurality Index of Ontario; SD = standard deviation.

### Sensitivity Analyses

When considering paediatric patients as individuals <24 years of age, our definition identified 326 child-focused psychiatrists in Ontario and 2032 adult-focused psychiatrists in 2023. When adjusting the percentage of outpatients seen in our definition to 30% and 70%, our definition identified 292 and 227 child-focused psychiatrists, respectively. Sensitivity analyses followed similar patterns to our main analysis; in all cases, child-focused psychiatrists were primarily female, younger, had fewer years since medical school graduation, and were less likely to be found in rural areas compared to adult-focused psychiatrists. They also were less likely to have smaller patient panels, particularly in lower supply areas.

## Discussion

In 2023, there were 259 psychiatrists who could be classified as child-focused psychiatrists based on ≥50% of their outpatients and/or inpatients being ≤18 years of age. Child-focused psychiatrists were more likely to be younger, female, and less likely to practice in rural areas compared to adult-focused psychiatrists. Child-focused psychiatrists also saw more new outpatients, had larger patient panels, and saw their patients less often compared to adult-focused psychiatrists. For both groups, psychiatrists practicing in regions where the average supply per capita of psychiatrists was well under the provincial average (low-supply areas) saw more patients and outpatients overall, were less likely to have smaller patient panels, and saw their patients for fewer visits than psychiatrists practicing in higher supply areas.

Regional analyses from previous studies indicate similar patterns in psychiatric practice; however, many of these studies are either outdated or analyse psychiatrists in general rather than child-focused psychiatrists.^5,16,27^ One such Canadian study using administrative data assessed psychiatrist characteristics and practice patterns by LHIN in 2009.^5^ Consistent with our findings, this study found that low-supply area psychiatrists saw, on average, a higher number of outpatients and were less likely to have smaller patient panels compared to higher supply areas.^5^ Further, psychiatrists in higher supply LHINs saw fewer outpatients overall, but provided care at a higher frequency. On average, these psychiatrists had about seven visits per patient per year compared to approximately four visits for psychiatrists in low-supply areas.^5^ Our results align with these patterns: across both groups, psychiatrists in lower supply areas saw more patients at a lower frequency compared to their counterparts in high-supply areas. However, among adult-focused psychiatrists, those in high-supply areas saw their patients almost twice as often as those in medium- or low-supply areas, with nearly 20% averaging more than 12 outpatient visits per patient per year. While child-focused psychiatrists in high-supply areas did see their patients more frequently than medium- and low-supply regions, there was not as much of a notable increase in frequency (approximately four visits per year compared to three visits). Further, all areas had zero to very few child-focused psychiatrists who saw their outpatients an average of >12 times per year. This provides evidence that child-focused psychiatrists may be practicing differently than their adult-focused counterparts, likely due to higher demand and lower supply. Even in high-supply regions, the need to serve a larger patient base may limit their ability to provide high-frequency follow-up, reflecting a system under strain.

Overall, there is a clear shortage of child-focused psychiatrists in Ontario, and this appears to influence how they structure their practice compared to adult-focused psychiatrists. The current supply rates are well under the recommendation of 1 psychiatrist for every 4,000 youth, and even under the general guidelines of 15 psychiatrists for every 100,000 citizens.^5,6^ Given the time-intensive nature of family-centered care in paediatric psychiatry, and the leadership role child-focused psychiatrists often assume within interdisciplinary mental healthcare teams, it is typically recommended that they manage smaller caseloads than psychiatrists treating adults.^8,9^ However, our findings did not reflect this expectation. Child-focused psychiatrists did not have smaller patient panels relative to their adult-focused counterparts. The difference in frequency of patient visits per year and the number of outpatients (new and overall) seen between groups is likely occurring due to the scarcity of psychiatrists. Faced with high demand and insufficient supply, child-focused psychiatrists appear unable to maintain smaller outpatient caseloads and are seeing higher numbers of outpatients (new and overall) at a lower frequency than their adult-focused colleagues.

The difference in outpatient visit frequency per year between groups may give insight to the type of care child-focused psychiatrists provide. Typical first-line treatment for children and adolescents is psychotherapy, usually involving 8–16 visits per year, followed by psychopharmacology, usually involving between 4–16 visits per year.^5,28^ Our results do not reflect these recommendations, particularly for psychotherapy. While some of our child-focused psychiatrists could operate consultation-only practices, another explanation could be that due to their high caseloads, psychiatrists may provide brief medication follow-ups or adjustments then refer their patients back to primary care for time-intensive treatment. If this is occurring, this could further amplify the difficulty in obtaining care for these individuals as primary care providers have reported a major barrier to providing mental healthcare is time constraints during visits.^29^ Future studies should investigate how child-focused psychiatrists use their appointment time to understand the implications of these models of care.

The structural and cultural differences between the child and adult mental healthcare systems may also influence patient visit frequency per year. Since child psychiatrists often assume a leadership role within interdisciplinary mental healthcare teams,^8^ this could place them in the role of a consultant. Therefore, child-focused psychiatrists in our study may be seeing their patients less often and handing off care to other team members. Contrastingly, adult mental health services are typically more autonomous and are quite siloed.^30,31^ While there has been a push for interdisciplinary mental healthcare teams across the country,^32^ the lack of interdisciplinary teamwork in adult mental healthcare may put all treatment responsibilities on the adult-focused psychiatrists. Adult providers may have to see their patients more frequently if they do not have a care team to provide support.

Lastly, the scarcity of child-focused psychiatrists seems to be magnified in certain regions of the province. We identified multiple areas where the average number of psychiatrists per capita was well under the already low overall provincial average, and physicians in these areas were even less likely to have small patient panels and saw patients less often compared to higher supply regions. These were regions with greater rural populations and/or Indigenous populations, reflecting regional inequities identified in other literature.^14,33^ The large standard deviations of the average number of patients (overall, inpatient and outpatient) imply a wide spread of patients seen among psychiatrists, further indicating practice patterns are not consistent across the province.

## Strengths & Limitations

The methods of our study are novel; to our knowledge, this is the first time administrative data has been used to create a definition of child-focused psychiatrists, or any subspecialty. Other specialties in Ontario could apply our methods to their field. Other provinces and territories could apply our methods to define child-focused psychiatrists in their jurisdictions to understand their specialist mental health care workforce and contribute to informing the Canadian mental healthcare system. Further, we used population-based, administrative data to provide important, baseline information for a specialist mental healthcare service that has not been reported since 1999. We updated this information to represent the current supply and distribution of child-focused psychiatrists in Ontario, which will be of interest to policymakers and providers interested in effectively and equitably planning Ontario's mental healthcare system.

This study has several limitations that should be considered. First, we are limited to the variables available within administrative health databases, which do not capture important sociodemographic characteristics such as gender identity, sexual orientation, race, and ethnicity, factors that are essential for assessing equity in the psychiatric workforce. Second, a limitation to administrative databases is missing data. In our regional analysis, 33 child-focused psychiatrists and 194 adult-focused psychiatrists did not have a LHIN associated with them and were excluded. Excluded physicians were more recent graduates of medical school and/or newer physicians to Ontario who saw fewer patients compared to those included (Supplement). We used LHINs in our analysis because we felt the granularity of the LHINs offered more insight into regional variation than the OH Regions. However, this may limit the application of our results to the current provincial health sectors and not reflect regional disparities seen by OH Region. Further, our sample may be biased to late career psychiatrists, and we may be missing identifying practice patterns of younger, early career psychiatrists who perhaps began clinical work after LHINs were replaced by OH Regions. Third, although the majority of physicians operate under a fee-for-service model, some receive compensation through alternative payment models such as salaries, hourly rates, capitation, or contractual arrangements.^34^ A 2016 report from the Canadian Institute for Health Information found that, while 99% of Ontario physicians billed at least partially through fee-for-service, 56% also used at least one alternative form of compensation.^35^ While some salaried doctors activities are recorded through shadow billings for administrative purposes, some clinical activity may be missed and excluded in this analysis. This is a very small number in general, so it is unlikely to bias our results; however, it is worthwhile to note.

## Conclusion

This study revealed that there is a low supply of child-focused psychiatrists in Ontario, and they are particularly scarce in rural regions. To keep up with demand, the evidence suggests that child-focused psychiatrists are seeing greater numbers of outpatients and have larger patient panels than their adult counterparts. They are also seeing their patients fewer times per year than adult-focused psychiatrists, and it is not known if patients are receiving follow-up care with primary care in lieu of continuous psychiatric care. These results offer valuable insights for policymakers and healthcare leaders seeking to address workforce gaps and geographic maldistribution of child-focused psychiatrists in Ontario. Finally, our novel, data-driven approach to defining child-focused psychiatrists offers a scalable framework that could be applied to other jurisdictions and subspecialties. Using similar methods, other provinces/territories could assess their psychiatric workforce, enabling a more comprehensive, national understanding of psychiatric care availability and unmet needs across Canada.

## Data Access

The dataset from this study is held securely in coded form at ICES. While legal data sharing agreements between ICES and data providers (e.g., healthcare organizations and government) prohibit ICES from making the dataset publicly available, access may be granted to those who meet pre-specified criteria for confidential access, available at www.ices.on.ca/DAS (email: das@ices.on.ca). The full dataset creation plan and underlying analytic code are available from the authors upon request, understanding that the computer programs may rely upon coding templates or macros that are unique to ICES and are therefore either inaccessible or may require modification.

## Supplemental Material

sj-docx-1-cpa-10.1177_07067437251408168 - Supplemental material for Defining, Locating, and Characterizing Psychiatrists who Primarily Treat Children and Adolescents and their Practices in Ontario: A Cross-Sectional Study: Définir, localiser et caractériser les psychiatres qui traitent principalement les enfants et les adolescents et leurs pratiques en Ontario : étude transversale Supplemental material, sj-docx-1-cpa-10.1177_07067437251408168 for Defining, Locating, and Characterizing Psychiatrists who Primarily Treat Children and Adolescents and their Practices in Ontario: A Cross-Sectional Study: Définir, localiser et caractériser les psychiatres qui traitent principalement les enfants et les adolescents et leurs pratiques en Ontario : étude transversale by Madison MacKinnon, Alene Toulany, Claire de Oliveira, Tea Rosic and Paul Kurdyak in The Canadian Journal of Psychiatry
