# Supplementary material for: Defining, Locating, and Characterizing Psychiatrists who Primarily Treat Children and Adolescents and their Practices in Ontario: A Cross-Sectional Study: Définir, localiser et caractériser les psychiatres qui traitent principalement les enfants et les adolescents et leurs pratiques en Ontario : étude transversale
Source: Can J Psychiatry. 2025 Dec 29;71(3):219–30. doi: 10.1177/07067437251408168 (PMC12747871; doi:10.1177/07067437251408168)

**Supplement**

**Table S1. Characteristics of Psychiatrists Included vs Excluded from LHIN Analysis due to missing data**

|  | **Child-focused Psychiatrists** | | | **Adult-focused Psychiatrists** | | |
| --- | --- | --- | --- | --- | --- | --- |
|  | **Excluded** | **Included** | **p** | **Not included** | **Included** | **p** |
| **No. of psychiatrists** | 33 | 226 |  | 194 | 1905 |  |
| **Sex (%)** |  |  | <0.001 |  |  | <0.001 |
| *Female* | 18 (54.5) | 135 (59.7) |  | 94 (48.5) | 877 (46.0) |  |
| *Male* | 15 (45.5) | 91 (40.3) |  | 100 (51.5) | 1028 (54.0) |  |
| **Canadian Medical School Graduate (%)^a^** | 0 (NaN)^b^ | 99 (65.6) | NaN ^b^ | <6 | 846 (62.9) | 0.746 |
| **Physician age (mean (SD))** | NaN (NA) ^b^ | 55.76 (9.34) | NA ^b^ | 61.80 (7.19) | 60.09 (11.46) | 0.739 |
| **Years Since Graduation (mean, SD)** | 9.00 (NA) | 24.76 (11.34) | NA ^b^ | 29.83 (13.01) | 28.51 (14.29) | 0.821 |
| **Years Since Graduation (%)** |  |  | <0.001 |  |  | <0.001 |
| *0-15* | 33 (100.0) | 52 (23.0) |  | 189 (97.4) | 407 (21.4) |  |
| *15-30^a^* | 0 ( 0.0) | 110 (48.7) |  | <6 | 690 (36.2) |  |
| *30y ^a^* | 0 ( 0.0) | 64 (28.3) |  | <6 | 808 (42.4) |  |
| **No. patients seen (mean, SD)** | 174.79 (136.23) | 291.62 (276.94) | 0.018 | 247.64 (267.67) | 337.74 (413.60) | 0.003 |
| **No. of inpatients seen (mean, SD)** | 41.03 (49.76) | 55.23 (78.77) | 0.316 | 75.47 (112.29) | 82.18 (139.08) | 0.516 |
| **No. of new inpatients seen (mean, SD)** | 39.91 (48.06) | 50.14 (69.92) | 0.417 | 72.30 (105.30) | 74.10 (123.84) | 0.846 |
| **No. of outpatients seen (mean, SD)** | 124.70 (118.88) | 226.81 (242.09) | 0.018 | 153.00 (215.05) | 230.08 (287.36) | <0.001 |
| **No. of New outpatients seen (Mean, SD)** | 111.33 (101.33) | 144.39 (161.40) | 0.254 | 137.16 (202.55) | 128.15 (190.75) | 0.533 |
| **No. of Inpatients (%)** |  |  | 0.082 |  |  | <0.001 |
| *0 ^a^* | <6 | 53 (23.5) |  | 48 (24.7) | 783 (41.1) |  |
| *>100* | 6 (18.2) | 41 (18.1) |  | 48 (24.7) | 519 (27.2) |  |
| *1-50* | 21 (63.6) | 95 (42.0) |  | 65 (33.5) | 429 (22.5) |  |
| *51-100^a^* | <6 | 37 (16.4) |  | 33 (17.0) | 174 ( 9.1) |  |
| **No. new Inpatients (%)** |  |  | 0.076 |  |  | <0.001 |
| *0 ^a^* | <6 | 53 (23.5) |  | 48 (24.7) | 790 (41.5) |  |
| *>100* | 6 (18.2) | 36 (15.9) |  | 48 (24.7) | 495 (26.0) |  |
| *1-50* | 21 (63.6) | 97 (42.9) |  | 67 (34.5) | 444 (23.3) |  |
| *51-100 ^a^* | <6 | 40 (17.7) |  | 31 (16.0) | 176 ( 9.2) |  |
| **No. of outpatients (%)** |  |  | 0.081 |  |  | <0.001 |
| *0 ^a^* | <6 | <6 |  | <6 | 20 ( 1.0) |  |
| *>100* | 15 (45.5) | 147 (65.0) |  | 84 (43.3) | 1143 (60.0) |  |
| *1-50 ^a^* | <10 | 19-29 |  | 69 (35.6) | 425 (22.3) |  |
| *51-100* | 10 (30.3) | 54 (23.9) |  | 29-39 | 317 (16.6) |  |
| **No. of new outpatients (%)** |  |  | 0.938 |  |  | 0.393 |
| *0 ^a^* | <6 | <6 |  | <6 | 87 ( 4.6) |  |
| *>100* | 14 (42.4) | 102 (45.1) |  | 76 (39.2) | 727 (38.2) |  |
| *1-50 ^a^* | <10 | 49-59 |  | 75 (38.7) | 746 (39.2) |  |
| *51-100* | 11 (33.3) | 70 (31.0) |  | 29-39 | 345 (18.1) |  |

^a^Cells collapsed due to small cell counts.

^b^No data available

**Figure S1.** Distribution of Paediatric Cases (defined as patients <19 years of age) Seen By Ontario Psychiatrists from 2013-2023


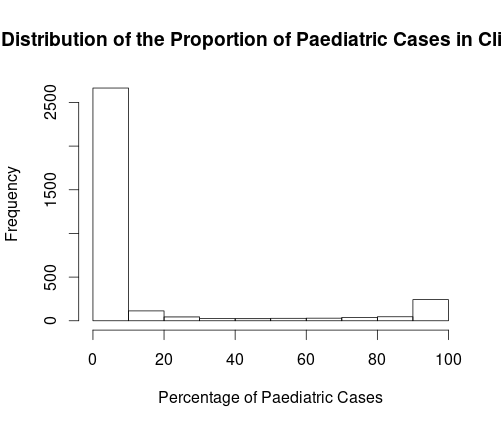

Supplement: sj-docx-1-cpa-10.1177_07067437251408168 - Supplemental material for Defining, Locating, and Characterizing Psychiatrists who Primarily Treat Children and Adolescents and their Practices in Ontario: A Cross-Sectional Study: Définir, localiser et caractériser les psychiatres qui traitent principalemen [file sj-docx-1-cpa-10.1177_07067437251408168.docx]
